# Diabetes and Growth of Tumour Transplants

**DOI:** 10.1038/bjc.1970.42

**Published:** 1970-06

**Authors:** Beryl M. A. Davies

## Abstract

Appreciable insulin occurred in transplants of a hamster and a mouse adrenal tumour. The hormone was measured by immunoassay. In hamster experiments, the weight of tumours grown in animals made mildly diabetic was five times less than those grown in controls. No insulin nor diabetic effect occurred in transplants of a mouse brain or stomach tumour.


### DIABETES AND GROWTH OF TUMOUR TRANSPLANTS

BERYL M. A. DAVIES

*From the Imperial Cancer Research Fund, Burtonhole Lane, London, N.W.7*

Received for publication January 21, 1970

**SUMMARY.—Appreciable insulin occurred in transplants of a hamster and a mouse adrenal tumour. The hormone was measured by immunoassay. In hamster experiments, the weight of tumours grown in animals made mildly diabetic was five times less than those grown in controls. No insulin nor diabetic effect occurred in transplants of a mouse brain or stomach tumour.**

THIS work was done to see if tumours, which arise from specific organs contain insulin or not and to investigate the effect of diabetes on the growth of such tumours.

Apart from insulin secreting fibrosoma of mediastinum in hypoglycaemic patients (Lowbeer, 1961), there appear to be little assay data on the insulin content of non-pancreatic tumours. With " generalized animal tumours ", four teams have reported decreased tumour growth in alloxan diabetic or partially depancreatized animals, thus indicating a controlling influence of insulin on this growth (Walker 256 carcinoma: Goranson and Tilser, 1955; Ingle, 1958; Garvie, 1968. Erlich ascites tumour: Jehl *et al.*, 1955). Apart from one paper using the Novikoff hepatoma (Goranson and Tilser, 1955), there seem to be no publications about the effect of diabetes on tumours arising from specific tissues. In the present work hepatomas were not used, since they are so vascular that any measurement of their insulin content would reflect both tumour and plasma insulin. As the mammary gland has a high fat content and insulin also affects fatty acid synthesis, tumours of this gland were not investigated.

### MATERIALS AND METHODS

Transplants of the following tumours were used:

*Hamster adrenal tumour FC/1584/60.*—This arose spontaneously in 1960 in a golden hamster in Dr. F. C. Chesterman's colony (London). It was an adrenal cortical carcinoma. Non-haemorrhagic and haemorrhagic portions (blood clots) of this tumour were collected separately. Results (insulin or tumour weight) refer to the non-haemorrhagic part only.

*Mouse adrenal tumour DA.*—Following the work of Dickie (1959), 1–2 day old CE strain mice were ovariectomized. In some animals 9–12 months later a unilateral adrenal tumour appeared. Only the insulin content of this tumour was measured.

*Mouse brain tumour* (Ependymoma A/22).—This appeared in 1948, 354 days after the implantation of a pellet of 20-methylcholanthrene into the brain (Professor H. M. Zimmerman, New York, U.S.A.).

*Mouse gastric adenoma G*328.—This appeared in 1949 after 20-methylcholanthrene had been injected into the wall of the glandular stomach in 1948 (Dr. H. Stewart, Bethesda, U.S.A.).

For each type, tumour weights and their insulin content were compared in control animals and in animals which had been treated with a diabetogenic agent, either one intracardiac (IC) or 3 sub-cutaneous (SC) injections of alloxan (which destroys pancreatic $\beta$ cells) or anti-insulin serum (AIS: antibodies to ox insulin raised in guinea-pigs). Tumour transplantation (IP or SC) was always on day 1. Alloxan diabetes in hamsters and mice was generally induced on day 1, but in experiment HA3 it was delayed until day 21. The use of AIS in small daily doses made the animals sub-diabetic. Drops of urine were tested daily or twice daily for glycosuria on Clinistix paper (Ames and Co.). Taking each Clinistix " + " as one point, the glycosuric score was kept for each alloxan-treated animal and these varied considerably. From the daily Clinistix results, graded doses of globin zinc insulin or protamine zinc insulin were given to prevent high mortality and yet to try to keep the animals still diabetic. Few of the animals were diabetic by the end of the experiments. Mortality of alloxan-treated hamsters was about 30%, while almost all the AIS hamsters and mice lived. No kidney lesions were seen at autopsy examination of 10 of our controls, 5 AIS-treated and 10 alloxan-treated hamsters, though occasionally in the last group there was some hyperaemic glomerular congestion (Dr. R. Vaughan). Hamster tumours were harvested at 6 or 9 weeks and mouse tumours at 7–8 days or 2½–3 weeks.

Tumours were collected in hexane, which was cooled by a cardice–acetone mixture and were stored at −18° C. Insulin was extracted by homogenizing and stirring with acid–alcohol. Unwanted protein was removed by precipitation at pH 8·5, and the insulin then precipitated with an alcohol–ether mixture (Best *et al.*, 1939; Morgan and Lazarow, 1965). It was dried over $P_2O_5$ in a vacuum desiccator and stored in this at +2° C. Before assay, the final extract was ground in a glass pestle and mortar, 0·006 M glycine/HCl buffer (pH 1–2) added and the solution diluted to the necessary strength using 0·04 M phosphate buffer (pH 7·4) containing 0·9% saline and 0·025% sodium ethyl mercurithiosalicylate.

Recovery of crude human or rat insulin added to acetone powdered pancreases of the same species was 69% and 80% respectively. A homogenate of a number of hamster adrenal tumours was made. Recovery of crude human insulin added to this was 96%.

Insulin was estimated by Hales and Randle's (1963) radioimmunoassay method (Type C), using the Radiochemical Centre's (Amersham, Bucks) reagent kit. The method is based on the competition between labelled and unlabelled (tumour) insulin to combine with a constant amount of guinea-pig anti-ox insulin serum. The complex formed is precipitated and counted. The reference standard was highly purified ox-insulin (six times recrystallized or chromatographically pure material, 23·8 biological i.u./g.). This was dissolved in the same way as the tumour extract. Our preliminary work on hamster and mouse serum had shown that insulin from these species could be estimated by these " reagents ". Each tumour extract was tested at five aliquot levels to check that there was always a proportionality between insulin found and the level of testing. Thus the calculation of insulin as milliunits (mu)/g. wet weight was not biased by the level selected for testing. If the binding power of the anti-ox insulin antibody is poor, as happened in preliminary work, this can destroy the proportionality relationship.

Parallel dose–response lines were obtained for the standard preparation and tumour extracts, while good agreement occurred between duplicate measurements of insulin in the same tumour extract.

### RESULTS

Results of insulin content and tumour weight are given in Tables I, II and III.

*Insulin in adrenal tumours (hamster and mouse): the impedance of adrenal tumour growth by diabetes (hamster)*

Estimations of insulin in batches of up to 46 pairs of normal hamster adrenals failed to detect this hormone. When four batches of 80–86 pairs were available, insulin in quantities of 0·19, 0·17, 0·04 and 0·10 milliunits (mu) of immunoreactive insulin/g. tissue were found. Hamster adrenal tumours (FC/1584/60) from control animals contained an average of 19 mu/g. wet weight. No insulin or negligible amounts of the hormone were present in tumours from alloxan or AIS injected hamsters (Table I).

TABLE I.—*Immunoreactive Insulin Content of Hamster and Mouse Adrenal Tumours, Mouse Brain Tumour and Mouse Stomach Tumour*

| Type of tumour | No. of estimations: Control | No. of estimations: Alloxan or AIS treated animals | Tumour insulin (mu/g. wet wt) Mean ± S.E. (range): Control | Tumour insulin (mu/g. wet wt) Mean ± S.E. (range): Alloxan or AIS treated animals |
|---|---|---|---|---|
| Hamster adrenal* | 1 tumour/estimation | | | |
| (FC/1584/60) | 4 | 5 | 18·3±8·3 (2·2–35·4) | Negligible† |
| | 5 | 6 | 18·7±4·3 (9·8–33·7) | Negligible |
| Mouse adrenal (DA) | 1 tumour/estimation | | | |
| | 1 | — | 58·5 | — |
| | 1 | — | 94·4 | — |
| Mouse brain (A/22) | 6–12 tumours/estimation | | | |
| | 7 | — | Negligible† | — |
| | 1 | — | 0·9 | — |
| | 1 | 1 | Negligible | Negligible |
| | 1 | 1 | Negligible | Negligible |
| Mouse stomach (G328) | 4–12 tumours/estimation | | | |
| | 13 | — | Negligible | — |
| | 1 | 1 | Negligible | Negligible |
| | 1 | 1 | Negligible | Negligible |

* The non-haemorrhagic part only of this tumour was estimated.
† Negligible = 0·30–0·02 mu/g. wet wt.

Two mouse DA adrenal tumours contained 58 and 94 mu insulin/g. wet wt (Table I). Owing to the slow growing nature of the tumour, no mouse diabetic experiments have been done.

In hamsters, diabetogenic conditions caused a failure of growth in the adrenal tumour transplants, as indicated by tumour weight. Thus, after six weeks, the transplants averaged 4–7 times less in weight than those grown in control animals. This result occurred both in conditions of sub-diabetes due to daily small injections of AIS or when alloxan diabetes was used. Expressing the glycosuric score as a percentage of the possible urinary glucose score, alloxan diabetes caused a 7–46

(mean of all experiments 25) score. The decreased growth of the tumour occurred whether the treatment began at the beginning or halfway through the growth period. Severe diabetes also caused loss of body weight. However, the decreased tumour growth seemed to be a specific effect of hypoinsulinism because it occurred in mild or sub-diabetic conditions (see alloxan experiments HA5 and HA6 and AIS experiments HA7, 8, 9, Table II), where body weight remained stable. The lower tumour weight of the tumours in experimental hamsters was always statistically significant. High glycosuric scores tended to be accompanied by large losses in tumour weight, but this was statistically significant only in experiment HA2 (Table II).

TABLE II.—*Effect of Alloxan or Anti-insulin Serum on Growth of a Hamster Adrenal Tumour (FC/1584/60)*

| Expt no. | No. of hamsters/group: Control | Expl | Mean gain in body wt† (g.): Control | Expl | Wt of tumour‡ (g.) ± S.E.: Control | Expl | Ratio of tumour Control/expl |
|---|---|---|---|---|---|---|---|
| 10 mg. alloxan (IC)/100 g. body wt | | | | | | | |
| HA1 | 4 | 5 | 20 | 9 | 2·1±0·9 | 0·5±0·2 | 4·2* |
| HA2 | 5 | 6 | 22 | 10 | 2·1±0·1 | 0·3±0·1 | 7·0*** |
| HA3§‖ | 6 | 11 | 4 | −8 | 3·4±1·3 | 0·7±0·2 | 4·9* |
| HA4 | 9 | 7 | 8 | −6 | 2·1±0·4 | 0·6±0·1 | 3·5* |
| 5 mg. alloxan (IC)/100g. body wt | | | | | | | |
| HA5 | 9 | 8 | 15 | 11 | 2·7±0·6 | 0·6±0·1 | 4·5** |
| HA6¶ | 5 | 5 | 27 | 26 | 3·5±1·2 | 0·9±0·4 | 3·9* |
| Anti-insulin serum†† | | | | | | | |
| HA7 | 5 | 10 | 12 | 11 | 5·6±0·8 | 1·0±0·3 | 5·6*** |
| HA8 | 7 | 10 | 9 | 7 | 3·9±1·0 | 0·8±0·2 | 5·0** |
| HA9‖ | 7 | 9 | 9 | 9 | 3·9±0·9 | 1·0±0·4 | 3·9** |

* $P = <0·05$, ** $P = <0·01$, *** $P = <0·001$.

† From day 1—day of autopsy.

‡ Refers to non-haemorrhagic portion of adrenal tumour.

§ Stomach tube feeding with a mixture of powdered pellets and milk.

‖ Alloxan was given on day 21: in other experiments given on day 1, the day of transplantation.

¶ Harvested at 9 weeks: in other HA experiments tumours collected at 6 weeks.

†† AIS was given from days 1–21 in experiments HA7 and HA8 and from days 21–41 in experiment HA9. Twice daily small doses were used which produced sub-diabetes. Single SC injection dose was 1/17 of one ampoule of K 5205 or Ref. 290365 (Burroughs Wellcome and Co.) for expt HA7 and 1/10,000 of one ampoule MR 51 for experiments HA8 and HA9.

It can be concluded that lack of insulin acts specifically to decrease the growth of hamster adrenal tumour FC/1584/60 and hence the tumour can be regarded as insulin-dependent.

*Absence of insulin in brain and stomach tumours: no diabetic effect on growth of these tumours*

Quantities of up to 4 g. normal mouse brain or up to 6 g. normal mouse stomachs failed to yield detectable immunoreactive insulin. Nor in estimations using up to 8 g. brain tumour or up to 5 g. stomach tumour obtained from control or experimental animals could insulin be measured (Table I). In three experiments for each type of tumour, alloxan diabetes did not produce a decrease in tumour weight over a 3-week period (Table III).

Efforts were made to establish that these results were " true negatives ", *i.e.* that the presence of insulin or a diabetic effect were not missed. Quantities of

TABLE III.—*Effect of Alloxan on Growth of a Mouse Brain Tumour (A/22) and a Mouse Gastric Tumour (G328)*

| Tumour type and expt No. | Tumour harvested (days) | No. of mice/group Control | No. of mice/group Expl | Mean gain in body wt (g.) Control | Mean gain in body wt (g.) Expl | Wt of tumour (g.) Control | Wt of tumour (g.) Expl | Ratio of tumour wt Control/Expl |
|---|---|---|---|---|---|---|---|---|
| 20 mg. alloxan (SC)/100 g. body wt on day 1 | | | | | | | | |
| Brain | | | | | | | | |
| MB1 | 21 | 10 | 14 | 1 | 1 | 1·2±0·2 | 1·8±0·3 | 0·7* |
| | | | | (Range 0–2) | | | | |
| 20 mg. day 1 + 10 mg. day 2 + 10 mg. day 3 of alloxan (SC)/100 g. body wt | | | | | | | | |
| Brain | | | | | | | | |
| MB2 | 21 | 10 | 15 | 1 | 1 | 1·2±0·2 | 1·5±0·2 | 0·8* |
| | | | | (Range 0–3) | | | | |
| 10 mg. alloxan (IC)/100 g. body wt on day 1 | | | | | | | | |
| Brain | | | | | | | | |
| MB3 | 21 | 8 | 11 | 2 | 1 | 1·1±0·2 | 1·8±0·2 | 0·6* |
| | | | | (Range −2 to +4) | | | | |
| 7·5 mg. alloxan (IC)/100 g. body wt on day 1 | | | | | | | | |
| Brain | | | | | | | | |
| MB4 | 7 | 6 | 17 | 4 | 0 | 0·3±0·2 | 0·2±0·1 | 1·5* |
| | | | | (Range −4 to +3) | | | | |
| 20 mg. day 1 + 10 mg. day 2 + 10 mg. day 3 of alloxan (SC)/100 g. body wt | | | | | | | | |
| Gastric | | | | | | | | |
| MS1 | 21 | 9 | 10 | 2 | 1 | 0·7±0·1 | 0·7±0·1 | 1·0* |
| | | | | (Range 0–3) | | | | |
| 10 mg. alloxan (IC)/100 g. body wt on day 1 | | | | | | | | |
| Gastric | | | | | | | | |
| MS2 | 21 | 3 | 12 | 7 | 4 | 0·2±0·1 | 0·2±0·1 | 1·0* |
| | | | | (Range 1–9) | | | | |
| Gastric | | | | | | | | |
| MS3 | 21 | 8 | 7 | 2 | 5 | 1·2±0·2 | 1·1±0·3 | 1·1* |
| | | | | (Range 0–8) | | | | |
| 7·5 mg. alloxan (IC)/100 g. body wt on day 1 | | | | | | | | |
| Gastric | | | | | | | | |
| MS4 | 8 | 6 | 12 | 4 | −0·2 | 0·3±0·2 | 0·2±0·1 | 1·5* |
| | | | | (Range −3 to +5) | | | | |

* *t* test not significant, $P = > 0\cdot05$.

tissue larger than those in the hamster adrenal experiments were used for insulin measurements. Sometimes the dose of alloxan was the same in both hamster adrenal tumour and mouse tumour experiments, *i.e.* 10 mg. alloxan/100 g. body wt IC on day 1. The mean glycosuria for the brain tumour experiments was 38 (range 25–57) and for the stomach tumour experiments 28 (range 17–41), expressed as a percentage of the possible score. Thus even diabetic conditions more severe than those induced in the hamsters, did not cause tumour weight loss. In case the later part of the growth in the 21 days experiments had escaped from the influence of the diabetogenic agent given on day 1 or days 1–3 and was masking a possible diabetic effect in the early stages, mouse and brain tumours were also collected at 7–8 days. Again, no influence of diabetes on tumour weight was found.

### DISCUSSION

Insulin has been extracted from adrenal tumours by the use of acid–alcohol and estimated by immunoassay. It is concluded that this insulin is active in the living tumours for the following reasons. The acid–alcohol method is the same used by Best *et al.* (1939) for his isolation of crude, biologically active insulin, so the hormone is unlikely to have undergone degradation during its extraction. The immunoassay is regarded as having a high specificity for insulin. Parallelism occurred between the results obtained in our immunoassays with aliquots of tumour extracts and with aliquots of standard highly purified insulin, known to have biological activity. This would indicate a lack of inhibitors and exclude the presence of interfering substances giving false positives in the tumour extracts. Steiner and his colleagues (Rubenstein, Steiner, Sooja Cho, Lawrence and Kirsteins, 1969) have found pro-insulin, precursor of insulin, present in weak concentrations in acid–alcohol extractions of bovine pancreas. This has about 20% of the biological activity of insulin and is able to react with some guinea-pig anti-bovine insulin sera. However, pro-insulin is converted to insulin in the islets with an *in vitro* half-life of about 1 hour. Final confirmation of our immunoassay results is given in the diabetes experiments. It has been demonstrated that insulin is important to the growth of the hamster adrenal tumour since diabetes impedes that growth. Conversely, there can be no effective insulin acting on the growth of the brain and stomach tumours investigated, since these are not affected by diabetes.

While the concentration of insulin is much lower in the hamster adrenal tumour than in the hamster pancreas (2·1 mu/g. wet wt; Sodoyez, *et al.*, 1968), the hamster adrenal tumour contained 19 mu/g. wet wt and the mouse between 58–94 mu/g. compared to 72 mu/g. in a pancreatic islet cell tumour (No. 2309) found in a male golden hamster treated with a 30 mg pellet of testosterone proportionate (Sodoyez *et al.*, 1967). The insulin would either have been produced by the adrenal or concentrated by it from the blood. Its biosynthesis has not yet been investigated but the tumour insulin level is about $10^3$ greater than the nomal plasma levels. No experiments on the possible steroid production of the tumour have been made.

Thirteen cases of human adrenal tumour associated with hypoglycaemia have been recorded (Symington, personal communication), so it is possible these produce insulin. Our finding of insulin in adrenal tumours of both mouse and hamster suggest it may be a characteristic of rodent adrenal tumours or of one type of adrenal tumour. Presumably there may be an alteration in adrenal glucose metabolism in the tumour-bearing animals. It may be pointed out that the normal adrenal gland utilized glucose both by glycolysis and via the hexose monophate (HMP) pathway (pentose shunt) (Dickens, 1955; Glock and McLean, 1954). The importance of the latter in tumour FC/1584/60 has not yet been investigated. Other workers have reported that the HMP pathway is under the control of insulin (Glock and McLean, 1954; Young, 1962; Beloff-Chain *et al.*, 1959), and that it is important in the metabolism of some " generalized tumours " (Beaconsfield and Liuzzi, 1963; Glock and McLean, 1954; Wenner and Weinhouse 1956; Sahasrabude, 1958; Sahasrabude *et al.*, 1960).

Negative results for the present of insulin and the effect of diabetes were obtained for mouse ependymoma A/22 and mouse stomach tumour G328. It would be interesting also to work on brain tumours arising from other neuroglial tissue, *i.e.* gliomas or astrocytomas. Also the present brain and stomach tumours have been passaged for about 20 years, so it would be preferable to continue work with recently induced tumours. Details of any " organ specific " tumours in hamsters or mice obtained by other workers would be most gratefully received.

I thank Dr. F. C. Chesterman, Dr. Lilian Pang and Dr. Stretton Young for histological help, and Dr. D. C. Roberts for the use of his Tumour Registry. Dr. G. Howard Smith, Wellcome Research Laboratories, kindly provided highly purified ox-insulin.

### REFERENCES

BEACONSFIELD, P. AND LIUZZI, A.—(1963) *Life Sci.*, **7**, 459.
BELOFF-CHAIN, A., CANTZARO, R., CHAIN, E. B., LONGINOTTI, C. L., MASI, I. AND POCCHIARI, F.—(1959) *Selected Sci. Papers Inst. Super. Sanita.*, **2**, 139.
BEST, C. H., CAMPBELL, J. AND HAIST, R. E.—(1939) *J. Physiol.*, **97**, 200.
DICKENS, F.—(1955) *Proc. 3rd. Int. Congr. Biochem.*, 170.
DICKIE, M.—(1959) See 'Transplantable and Transmissible Tumours of Animals' in 'Atlas of Tumour Pathology', Section XII, STEWART, H. L., SNELL, K. C., DUNHAM, L. J. AND SCHLYEN, S. M., Armed Forces Institute of Pathology, Washington, D.C., p. 272.
GARVIE, W. H. H.—(1968) *Br. J. Cancer*, **32**, 128.
GLOCK, G. E. AND MCLEAN, P.—(1954) *Biochem. J.*, **58**, 171.
GORANSON, E. S. AND TILSER, G. J.—(1955) *Cancer Res.*, **15**, 626.
HALES, C. N. AND RANDLE, P. J.—(1963) *Biochem. J.*, **88**, 137.
INGLE, D. J.—(1958) *Endocrinology*, **62**, 78.
JEHL, J., MAYER, J. AND MCKEE, R. W.—(1955) *Cancer Res.*, **15**, 341.
LOWBEER, L.—(1961) *Am. J. clin. Path.*, **35**, 233.
MORGAN, C. R. AND LAZAROW, A.—(1965) *Diabetes*, **14**, 669.
RUBENSTEIN, A. H., STEINER, D. F., SOOJA CHO, B. S., LAWRENCE, A. AND KIRSTEINS, B.—(1969) *Diabetes*, **18**, 598.
SAHASRABUDE, M. B.—(1958) *Nature, Lond.*, **182**, 163.
SAHASRABUDE, M. B., NERURKAR, M. K., NARUKAR, M. V., TILAK, B. D. AND BHAVSAR, M. D.—(1960) *Br. J. Cancer*, **14**, 547.
SODOYEZ, J. C., LUYCKX, A. S. AND LEFEBVRE, P. J.—(1967) *Diabetes*, **16**, 415.
SODOYEZ, J. C., SODOYEZ-GOFFAUX, WHITTY, A. AND FOA, P. P.—(1968) *Diabetes*, **17**, 343.
WENNER, C. E., AND WEINHOUSE, S.—(1956) *J. biol. Chem.*, **222**, 399.
YOUNG, F. G.—(1962) *Proc. R. Soc. Ser. B.*, **157**, 1.


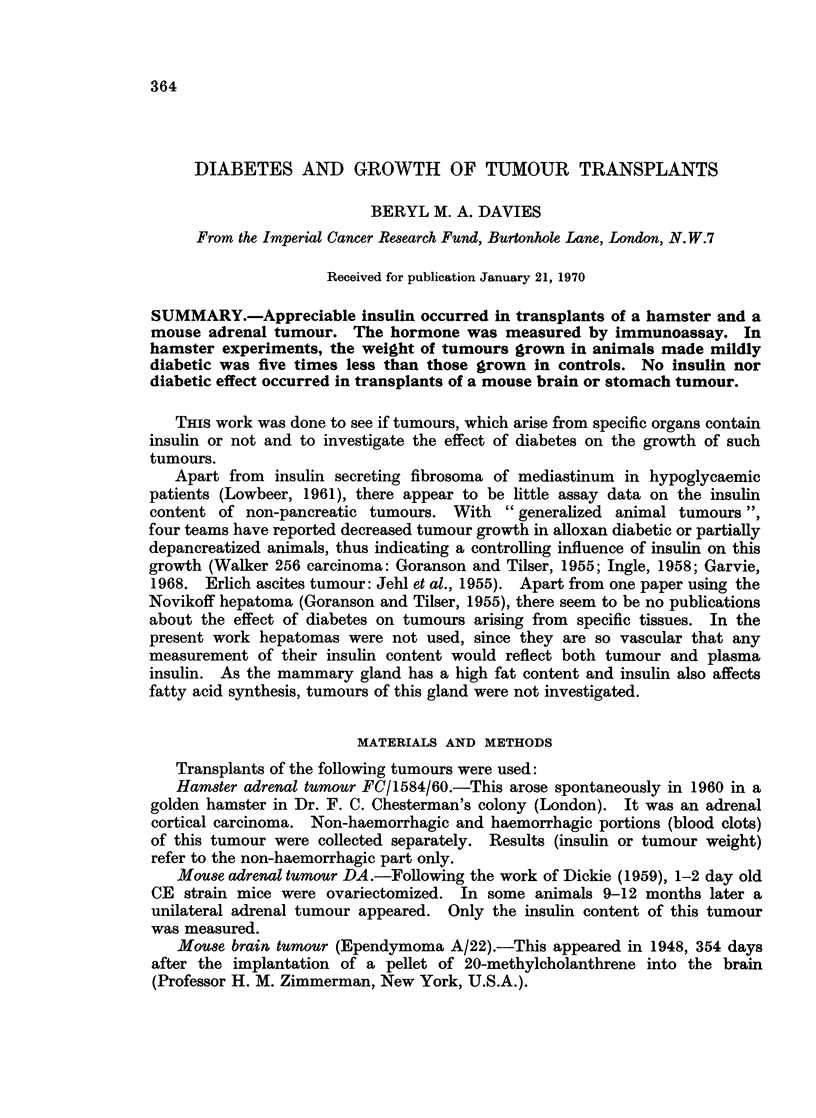

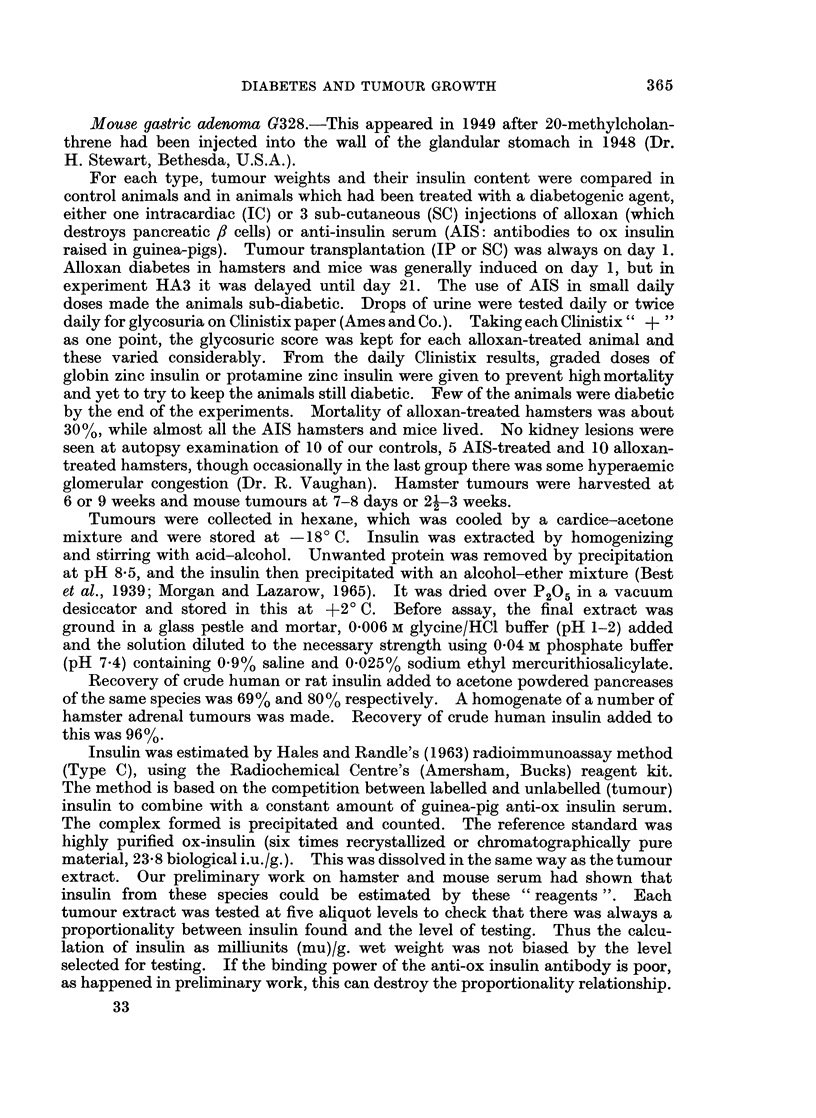

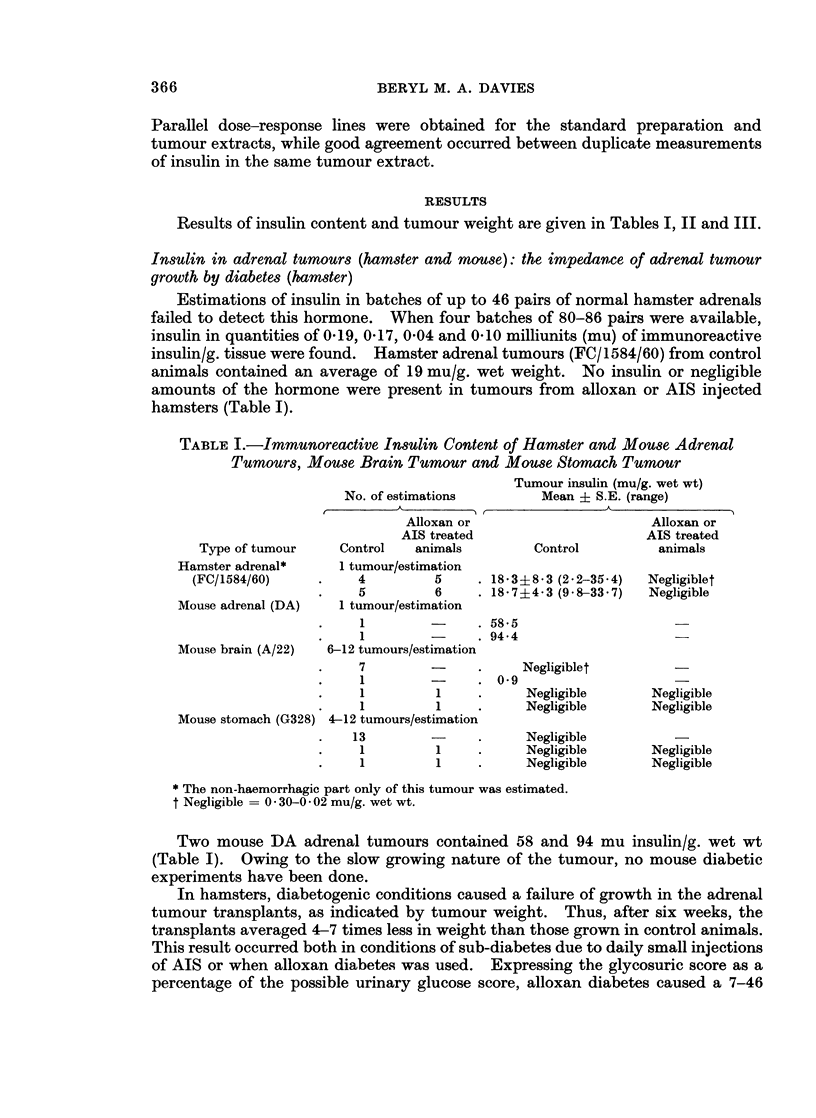

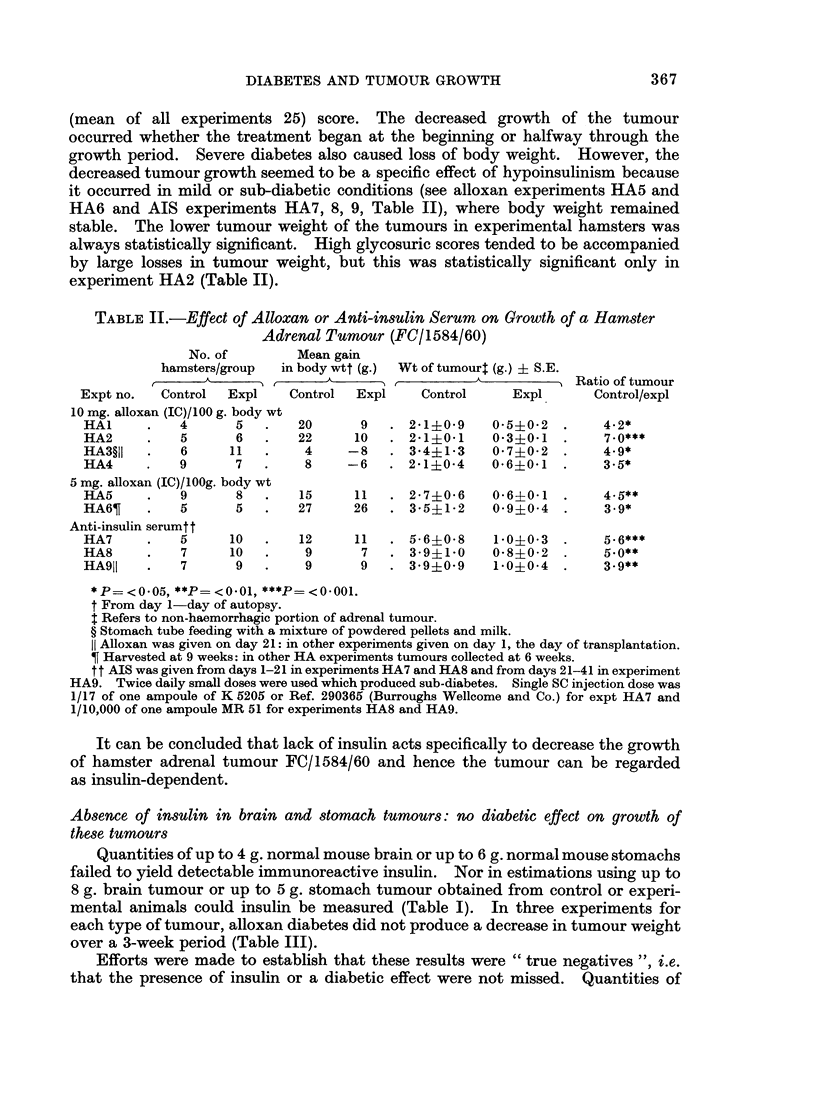

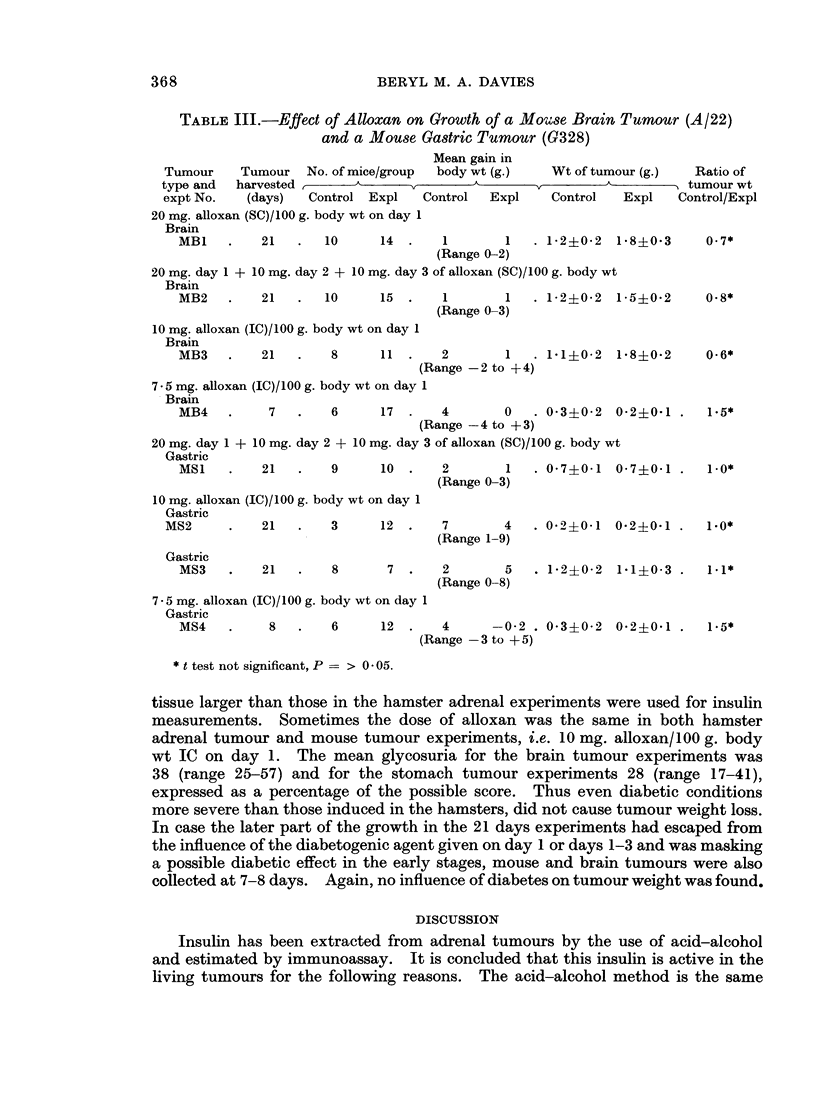

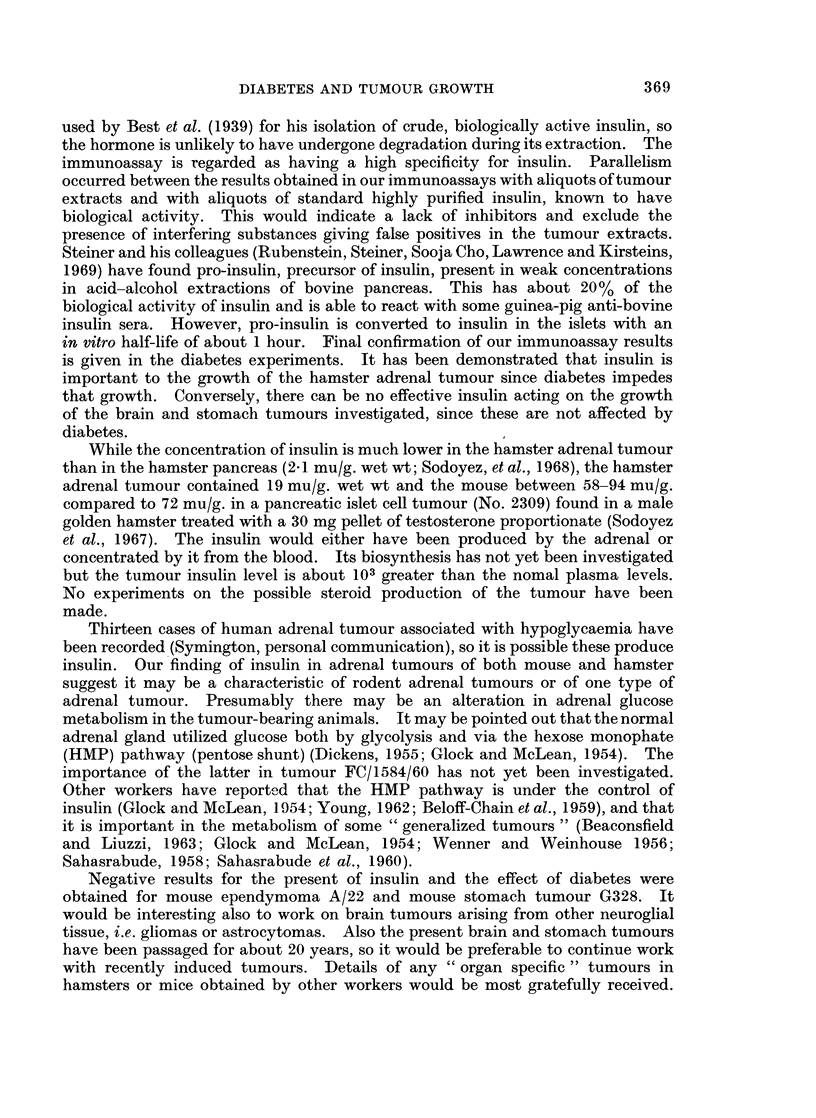

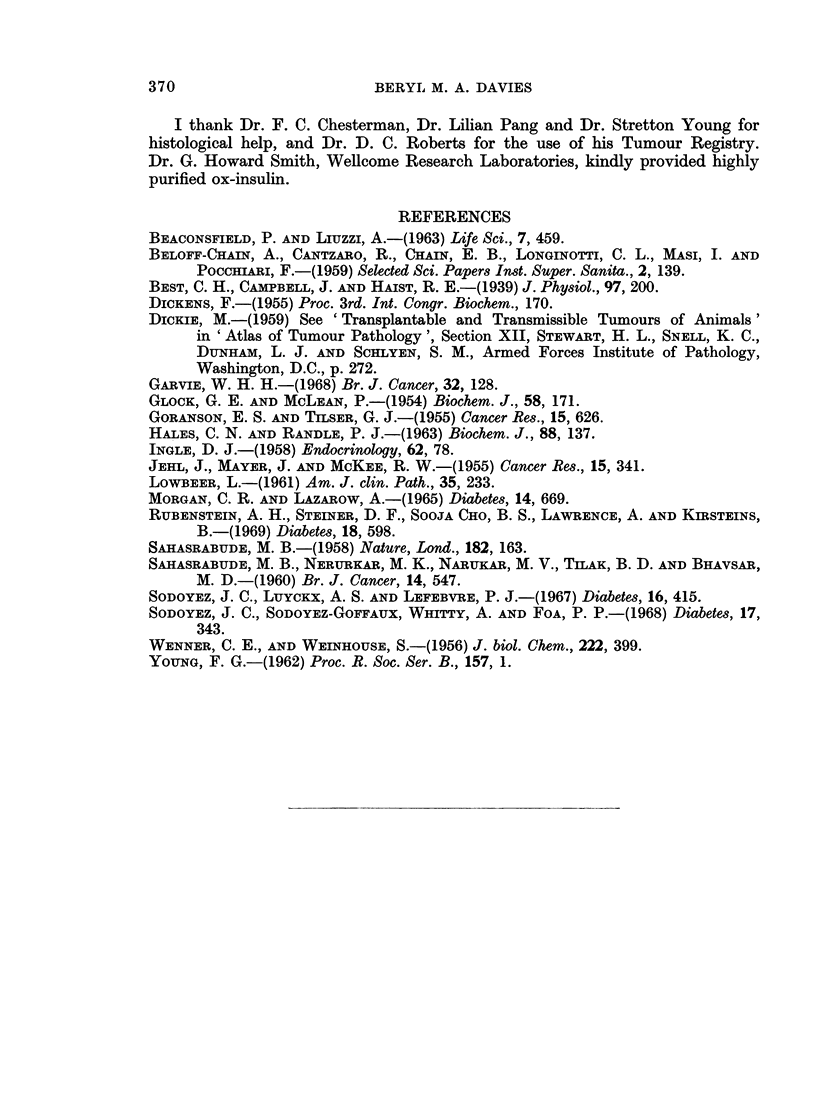


## References

[OCR_00438] BEACONSFIELD P., LIUZZI A. (1963). The significance of glucose metabolism via the pentose phosphate pathway in animal tissue.. Life Sci.

[OCR_00444] Best C. H., Campbell J., Haist R. E. (1939). The effect of anterior pituitary extracts on the insulin content of the pancreas.. J Physiol.

[OCR_00457] HALES C. N., RANDLE P. J. (1963). Immunoassay of insulin with insulin-antibody precipitate.. Biochem J.

[OCR_00461] LOWBEER L. (1961). Hypoglycemia-producing extrapancreatic neoplasms. A review.. Am J Clin Pathol.

[OCR_00463] Morgan C. R., Lazarow A. (1965). Immunoassay of pancreatic and plasma insulin following alloxan injection of rats.. Diabetes.

[OCR_00465] Rubenstein A. H., Steiner D. F., Cho S., Lawrence A. M., Kirsteins L. (1969). Immunological properties of bovine proinsulin and related fractions.. Diabetes.

[OCR_00471] SAHASRABUDHE M. B., NERURKAR M. K., NARURKAR M. V., TILAK B. D., BHAVSAR M. D. (1960). Inhibition of tumour growth by interference of hexose mono-phosphate pathway. Synthesis and anticancer properties of thiophene 2 : 5 dicarboxylic acid.. Br J Cancer.

[OCR_00475] Sodoyez J. C., Luyckx A. S., Lefebvre P. J. (1967). Biological properties of a transplantable islet-cell tumor of the golden hamster. II. Insulin content of the tumor and some metabolic characteristics of the tumor-bearing animals.. Diabetes.

[OCR_00481] WEINHOUSE S., WENNER C. E. (1956). Metabolism of neoplastic tissue. IX. An isotope tracer study of glucose catabolism pathways in normal and neoplastic tissues.. J Biol Chem.

